# Ala97Ser transthyretin amyloidosis-associated polyneuropathy, clinical and neurophysiological profiles in a Thai cohort

**DOI:** 10.1186/s12883-021-02243-3

**Published:** 2021-05-22

**Authors:** Nath Pasutharnchat, Chamaiporn Taychargumpoo, Yongkasem Vorasettakarnkij, Jakkrit Amornvit

**Affiliations:** 1 Division of Neurology, Department of Medicine, Faculty of Medicine, Chulalongkorn University, Bangkok, Thailand; 2 King Chulalongkorn Memorial Hospital, Thai Red Cross Society, Bangkok, Thailand; 3 Hospital for Tropical Diseases, Faculty of Tropical Medicine, Mahidol University, Bangkok, Thailand; 4 Department of Medicine, Faculty of Medicine, Chulalongkorn University, Bangkok, Thailand

**Keywords:** hereditary transthyretin amyloidosis-associated polyneuropathy, transthyretin, Ala97Ser, Thais, neurophysiological study, autonomic neuropathy, carpal tunnel syndrome, quantitative sensory test, composite autonomic severity score

## Abstract

**Background:**

Ala97Ser transthyretin amyloidosis-associated polyneuropathy (ATTRA97S-PN) is a rare form of inherited polyneuropathy, usually manifesting with late-onset (> 50) progressive polyneuropathy. This mutation is mostly prevalent in Taiwanese and Han-Chinese individuals. The aim of this study was to describe the clinical and comprehensive neurophysiological profiles of ATTRA97S-PN in Thai patients.

**Methods:**

The clinical profiles and serial neurophysiologic studies (nerve conduction study (NCS), quantitative sensory test (QST), and comprehensive autonomic function test (AFT)) of symptomatic ATTRA97S-PN patients who had been followed-up at King Chulalongkorn Memorial Hospital during 2010–2020 were retrospectively reviewed.

**Results:**

Nine symptomatic patients (55.6 % were male) from four unrelated families were included. All were Thais of mixed Thai Chinese descent. The mean age of onset was 48.3 (32–60) years. The mean age at diagnosis was 54.8 (33–66) years. Three patients developed early-onset (< 40y) polyneuropathy. The mean Neuropathy Impairment Score was 41.33 (10–92) at diagnosis. Sensory (9/9) and autonomic (9/9) neuropathies were more frequent than motor neuropathy (5/9), which appeared in the late stage of disease. Hypoesthesia in the feet, and gastrointestinal autonomic symptoms were frequently reported as the initial symptoms. The course of neuropathy progressed over years to decades. The worsening of neuropathy tended to progress faster once motor nerves were affected in both clinical and neurophysiological aspects. Concurrent cardiac amyloidosis was found in 6/9 patients. NCS showed length-dependent sensorimotor axonal polyneuropathy in 5/9 patients, and median neuropathy at the wrist (mostly bilateral) in 7/9 patients. QST showed abnormalities in the vibratory detection threshold, the cold detection threshold and the heat pain sensation in 8/9, 8/9 and 7/7 tested patients, respectively. AFT results were abnormal in all. The mean composite autonomic severity score was 5 (3–9).

**Conclusions:**

This clinical study is the first of ATTRA97S-PN in Thai patients. The mixed polyneuropathy-cardiopathy phenotype was the most common manifestation. In this cohort, the age of onset was lower, and the course of neuropathy was relatively longer, than that in previous studies. Some patients may develop early-onset polyneuropathy. This mutation has not yet been documented in any population other than Han Chinese-related populations, probably suggesting a founder effect. Further studies are warranted.

**Supplementary Information:**

The online version contains supplementary material available at 10.1186/s12883-021-02243-3.

## Background

Hereditary transthyretin amyloidosis (ATTRv) is a rare autosomal dominant, multisystem disorder. This disease is caused by mutations in the *TTR* gene, resulting in the deposition of misfolded transthyretin protein as amyloid fibrils in various organs [1, 2]. The commonly affected organs are the peripheral nerves and the heart. Others include the leptomeninges, eyes, gastrointestinal tract, and kidneys [2]. If left untreated, the disease is progressive and becomes life-threatening. To date, over 140 pathogenic mutations in the *TTR* gene have been found [3]. The heterogeneity of phenotypic expression, for example, main organ involvement, age of onset, and progression, has been shown among individuals with different *TTR* mutations, and in individuals of different ethnic groups, and geographic distributions. Phenotypic heterogeneity within the same pedigree is also not unusual [1, 2].

Hereditary transthyretin amyloidosis-associated polyneuropathy (ATTRv-PN) is a leading presentation in ATTRv. It is characterized by chronically progressive sensory, motor, and/or autonomic polyneuropathy, often with concurrent cardiac dysfunctions. TTRV30M, the most frequent mutation in ATTRv-PN, has been found in 47.6 % of cases worldwide [4]. This pathogenic variant has been mainly reported in Europe and some Asia-Pacific countries [2, 4, 5]. It could be present as early-onset (late 20 to 40 s) disease in endemic or late-onset (> 50 years) disease in non-endemic areas [5]. Being mostly prevalent in Taiwan, TTRA97S *(p. TTRA117S)*, usually manifests with a late-onset progressive polyneuropathy [4, 6]. Haplotype analyses, performed in 15 Han-Taiwanese families with TTRA97S, suggested the presence of a founder effect [6]. Clinical data of ATTRA97S-PN were mainly derived from Taiwanese patients [6–10]. In this study, the authors described the clinical and serial neurophysiological profiles of ATTRA97S-PN in a Thai cohort.

## Methods

### Patients

The electronic medical records of all symptomatic patients with ATTRA97S-PN, who had been followed-up at King Chulalongkorn Memorial Hospital during 2010–2020, were reviewed. Symptomatic patients were defined as patients having symptoms and signs of sensory, motor and/or autonomic polyneuropathy, which were not the result of other etiologies (e.g., diabetes mellitus, nutritional deficiency, thyroid disorders, drug and intoxication, alcohol abuse, and chronic inflammatory diseases). All patients had undergone genetic studies, showing a heterozygous variant, NM_00371.3: c.349G > T (p. Ala117Ser) in the *TTR* gene. All genetic studies were done at a commercial laboratory (Invitae Corporation, California, United States).

Demographic data, clinical data and neurophysiological findings were retrospectively reviewed. Composite Autonomic Symptom Score-31 (COMPASS-31) questionnaire, which has been employed in our clinical practice, was also obtained. The Neuropathy Impairment Score (NIS), a composite score of clinical impairment (weakness, reflex loss and sensory deficit), was reviewed. Higher NIS scores reflect greater deficits (score range, 0-244) [11].

The results of electrocardiogram (EKG)/Holter monitoring, echocardiogram, cardiac magnetic resonance imaging (MRI) and myocardium scintigraphy with the bone avid tracer ^99m^technitium pyrophosphate were reviewed. Cardiac amyloidosis was defined by following the expert consensus recommendations [12, 13]. The assessment of systemic involvement, including the involvement of the CNS, eye and kidneys, was mainly based on, history, complete physical, neurological and ophthalmological examinations, laboratory tests (urinalysis, renal function tests) and available imaging studies.

Data were described, compared among individuals, and compared with previous studies. This study was approved by the institutional review board. All methods were performed in accordance with the relevant guidelines and regulations. All patients had signed written informed consents.

### Neurophysiological studies

In this study, three neurophysiological studies, including (1) nerve conduction study (NCS) and electromyography (EMG), (2) quantitative sensory test (QST) and (3) autonomic function test (AFT) were done.

1. NCS and EMG were carried out with Viking on Nicolet EDX (Viking software) using standard techniques and our laboratory reference values. Protocols of the study are shown in additional file 1.
2. QST was done with the CASE IV™ system (WR Medical Electronics Co. USA). Tests included evaluations of the vibratory detection threshold (VDT), the cold detection threshold (CDT) and the heat pain sensation (HPS). Tests were done in the left upper and left lower limbs. The testing sites for the VDT were 1, at the midline of the great toe between the base of the nail and the first joint, and 2, at the base of the nail of the index finger. Testing sites for the CDT and HPS were the dorsal surfaces of the hand and foot. Abnormality was defined when measured values were > 95th percentile or < 5th percentile of the reference values.
3. AFT was performed with a full autonomic testing lab (WR Medical Electronics Co. USA). Tests included (1) Quantitative sudomotor axonal reflex test (QSART), (2) Heart rate variability to deep breathing, 3 Valsalva maneuver and 4. Tilt table test. Results were derived to calculate the composite autonomic severity score (CASS), according to published criteria [14]. In this study, CASSs of 2–3, 4–6 and 7–10 were defined as mild, moderate, and marked severities, respectively [15].

QST and AFT were performed, following their standard protocols.

## Results

### Clinical profiles

Nine symptomatic patients (55.6 % were male) from four unrelated families were included. The demographic data and clinical profiles of these patients were shown in Table 1. All were Thais of mixed Thai Chinese descent. Two lived in metropolitan Bangkok and 7 resided in the central vicinities of Thailand. Family history was reported in all patients (All patients in families A and B had family history of amyloid polyneuropathy or sudden and unexplained cardiac arrest. Patients C1 and D1 had first degree relatives who had had an undetermined cause of polyneuropathy and developed sudden and unexplained cardiac arrest.). The mean age of onset was 48.3 years. The mean age at diagnosis was 54.8 years. The course of disease duration ranged from 4 to 22 years. Overall, sensory (9/9) and autonomic (9/9) neuropathies were more frequent than motor neuropathy (5/9). At diagnosis, the mean NIS was 41.33. Seven patients reported clinical symptoms of carpal tunnel syndrome (CTS). Two patients developed CTS five years prior to sensory polyneuropathy. Hypoesthesia starting in the feet was the most frequent sensory complaint, found in 7/9 patients. Two patients had distal hyperesthesia and hyperalgesia. No patient had allodynia. Two patients (patients A1 and A4) had sensory symptoms starting in the hands. In all patients with motor neuropathy, weakness and muscular atrophy symmetrically affected distal muscles more than proximal muscles of the limbs. No patient had cranial neuropathy.

**Table 1:** Clinical profiles of 9 symptomatic ATTRA97S-PN

Family/ Patient	Sex	Age of onset (year)	Age at diagnosis (year)	Neuropathy at presentation	Neuropathic pain	Fiber type dysfunction of sensory nerve	Motor	Autonomic symptoms	COMPASS-31	Cardiopathy	Systemic
A1	M	52	60	S, M, **A**, **CTS**	*-*	Large, Small	U,L	**GI**, GU, **OH**	NA	P^a^ (CD, NYHA-III)	WR, dysphagia
A2	F	60	66	**S**, M, **A**, CTS	-	Large, Small	L	**GI**, GU, OH, **SU**	27.43	P^b^ (NYHA-I)	WR
A3	F	56	59	**S, A**, CTS	-	Large, Small	-	**GI, OH**	36.42	P^b^ (NYHA-I)	-
A4	F	52	55	S, **A**, **CTS**	P	Small	-	**GI**, SU	18.94	Ab^a^	-
A5	M	32	33	S, **A**	*-*	Large, Small	-	**OH**	20.46	Ab^b^	-
B1	M	58	66	**S**, M, A, CTS	P	Large, Small	U, L	GI, OH	NA	P^a^ (CD, NYHA-III)	WR
B2	M	32	36	S, **A**	*-*	Large, Small	-	**GI**	23.36	Ab^b^	-
C1	F	58	66	**S**, M, **A**, CTS	-	Large, Small	U, L	GI, GU, **OH**, SU	42.56	P^b^(AF, NYHA-II)	WR, MGUS
D1	M	35	52	S, M, **A**, CTS	-	Large, Small	U, L	**GI**, GU, OH, **SU**	33.68	P^c^ (CD, NYHA-II)	WR

**Bold**: initial symptoms, *M* male, *F* female, *S* sensory, *M* motor, *A* autonomic, *CTS* carpal tunnel syndrome, *U* upper limbs (excluding carpal tunnel syndrome), *L* lower limbs, *GI* gastrointestinal, *GU* genitourinary, *OH* orthostatic intolerance/hypotension, *SU* sudomotor, *COMPASS-31* The composite autonomic symptom score-31 questionnaire, *NA* non applicable, *P* present, *Ab* absent, *CD* conduction defects, *AF* atrial fibrillation, *NYHA* New York Heart Association functional classification (at the last follow-up), *WR* weight reduction, *MGUS* monoclonal gammopathy with undetermined significance,

^a^ Evident by EKG/Holter monitoring and echocardiogram, ^b^ Evident by electrocardiogram/Holter monitoring, echocardiogram and cardiac magnetic resonance imaging, ^c^ Evident by electrocardiogram/Holter monitoring, cardiac magnetic resonance imaging and myocardial scintigraphy with bone avid tracer ^99m^technetium pyrophosphate

A5 is the son of A1. B2 is the son of B1

Gastrointestinal symptoms were the most frequent autonomic complaints (8/9), followed by orthostatic intolerance (7/9), sudomotor dysfunction (4/9) and genitourinary symptoms (4/9). COMPASS-31 showed a mean score of 28.98. Autonomic symptoms, notably gastrointestinal symptoms, were frequently reported as the initial symptoms (Table 1. **Bold**). Four patients who underwent gastrointestinal endoscopies had unremarkable findings.

Cardiac involvement was found in 6/9 patients. Excluding orthostatic hypotension, four patients had at least one of the cardiac-related symptoms and signs. Patients A2 and A3 had no cardiac symptoms, but evidence of cardiac involvement was shown by cardiological imaging studies. Among 4 patients with cardiac symptoms, all symptoms developed after neuropathy. Patient D1 had a permanent cardiac pacemaker implanted for a complete heart block. Significant weight reduction was found in five patients. The maximum loss was 22 % of the body weight over two years. None had clinical evidence of central nervous system involvement, ocular involvement, or renal dysfunction. Amyloid deposition in the sural nerve was found in 1/1 tested patient. One patient had monoclonal gammopathy of undetermined significance (MGUS).

Total follow-up periods ranged from 3 to 7 years. The neuropathic conditions of five patients who had sensory, motor, and autonomic polyneuropathy at presentation worsened more rapidly than those of patients who had preserved motor nerves at presentation (mean rate of change in the NIS was 9.89 points/year). Three patients (patient A1, B1 and C1) had progressed from familial amyloid polyneuropathy (FAP) stage 2 to 3 in 3–5 years. Two patients died of cardiac amyloid-related conditions. Four patients, who had preserved motor nerve functions at presentation, did not have a significant worsening of polyneuropathy during the follow-up time up to 4 years (mean rate of change in the NIS was 0.25 points/year). One had a worsening of CTS. Three of 9 patients (patients A2, C1 and D1) had received diflunisal (mean rate of change in the NIS was 6.43 points/year). No patient in this cohort received other TTR tetramer stabilizers.

### Neurophysiological profiles

NCS/EMG and QST were conducted in all. The AFTs were completely performed in seven patients. Follow-up studies were also obtained at least once, except for patient C1. Table 2 shows the results of neurophysiological studies at the first evaluation and at the patients’ last visits. NCS/EMG showed a length-dependent large fiber axonal sensory and motor polyneuropathy in 5/9 patients and median neuropathy at the wrist in 7/ 9 patients. In all, except one patient with congenital transverse deficiency of the left hand, CTS was bilateral. All cases of CTS were at least moderate to severe. Abnormal VDT, CDT and HPS were detected in 8/9, 8/9 and 7/7 tested patients, respectively. In their first evaluations, the results of AFTs were abnormal in all patients with a mean CASS of 5 (3–9). Abnormal sudomotor, cardiovagal and adrenergic functions were found in 7/7, 7/7 and 6/7 tested patients, respectively. At the first evaluation, at least 2/3 neurophysiological tests were abnormal in all symptomatic patients.

**Table 2:** Neurophysiological profiles of symptomatic ATTRA97S-PN at the first and the last evaluations

Family / Patient	Study	NIS	SNCS		MNCS			CTS	QST			CASS	Follow-up Time (years)
			L ulnar SNAP amplitude (µV)	L sural SNAP amplitude (µV)	L ulnar CMAP amplitude (mV)	L fibular CMAP amplitude (mV)	L tibial CMAP amplitude (mV)		VDT LUL/LLL	CDT LUL/LLL	HPS LUL/LLL		
***Patients with sensory and autonomic polyneuropathy at presentation***.													
A3^a^	1st	18	20.9^a^	11	10^a^	3.2	14.3	Severe, R	+^a^/+	+^a^/+	+^a^/+	5	4
	Last	20	22^a^	10	10.3^a^	3.4	10.6	Severe, R	+^a^/+	+^a^/+	+^a^/ +	5	
A4	1st	10	20	11	8.3	3.2	15.7	Mod, B	-/ -	-/+	+/+	3	4
	Last	12	22	9.1	10.2	4.2	16.5	Mod, B	-/ -	-/+	+/+	3	
A5	1st	10	27	9.1	10.8	6	12.7	A	-/+	-/-	+/+	4	3
	Last	10	30	10.7	10.9	6.2	10.7	A	-/+	-/-	+/+	3	
B2	1st	12	16.8	11.3	10.4	3.3	11.1	A	+/++	-/+	NA	3	3
	Last	12	12.7	11.3	10.5	3.9	8.1	A	+/++	-/+	NA	4	
***Patients with sensory, motor and autonomic polyneuropathy at presentation.***													
A1	1st	84	NR	NR	2.3	NR	0.8	Severe, B	++/++	++/++	NA	NA	6 (death)
	Last	145	NR	NR	NR	NR	NR	U	NA	NA	NA	NA	
A2	1st	30	12.6	4.5	8.2	1.4	3.9	Severe, B	++/++	-/+	-/+	5	4
	Last	60	4.7	NR	5	0.5	1.3	Severe, B	++/++	+/++	+/++	7	
B1	1st	92	NR	NR	1.5	NR	NR	Severe, B	++/++	++/++	++/++	NA	3 (death)
	Last	152	NR	NR	NR	NR	NR	U	++/++	++/++	++/++	NA	
C1	1st	72	8	NR	3.8	NR	4.2	Severe, B	++/++	++/++	+/++	9	5
	Last	109	4.4	NR	2.5	NR	2.4	Severe, B	++/++	++/++	++/++	NA	
D1	1st	44	4.4	4	6.5	1.9	6	Severe, R Mod, L	++/++	++/++	++/++	6	5
	Last	66	NR	NR	6	1.2	3.5	Severe, R Mod, L	++/++	++/++	NA	7	

*NIS* neuropathy impairment score, *SNCS* sensory nerve conduction study, *MNCS* motor nerve conduction study, *L* left, *SNAP* sensory nerve action potential, *µv* microvolts, *CMAP* compound muscle action potential, *mV* millivolts, *CTS* carpal tunnel syndrome, *QST* quantitative sensory test, *VDT* vibratory detection threshold, *CDT* cold detection threshold, *HPS* heat pain sensation, *LUL* left upper limb, *LLL* left lower limb, *CASS* composite autonomic severity score, *R* right, *Mod* moderate, *B* bilateral, *A* absent, *NR* no response, *U* undetermined, +: >95th to 99th percentile, ++: >99th percentile, -: >5th to < 95th percentile, *NA* non applicable,

^a^ patient A3 had congenital transverse deficiency of the left hand. (Studies were done on the right side.),

Reference values: SNCS: ulnar ≥ 10µV, sural ≥ 6µV, MNCS: ulnar ≥ 6mV, fibular ≥ 2mV, tibial ≥ 5mV

## Discussion

The clinical presentation of ATTRv-PN varies among patients with different genetic, ethnic, and geographic backgrounds. Whatever is known in ATTRv-PN has been mainly gathered from studies of ATTRV30M. ATTRA97S has much lesser data, but usually shows a mixed polyneuropathy-cardiopathy phenotype of late-onset manifestation [6–10, 16]. Cardiopathy tended to become symptomatic after neuropathy in most cases [16]. ATTRA97S is mostly endemic in Taiwan (Han-Taiwanese) with male predominance [7–10]. It is also a common *TTR* mutation in mainland China (Han-Chinese), particularly in southern China [17–20]. One study from Malaysia reported ATTRA97S-PN in nine Chinese Malaysian patients [21]. In fact, this mutation has never been reported in other populations so far. Our study first described ATTRA97S-PN in Thai patients. The main clinical features in Thai patients were generally comparable to those in previous reports, however some additional characteristics were found.

Similar to previous reports, most of our patients also had a mixed polyneuropathy-cardiopathy phenotype. The core neuropathic manifestations were (1) symmetrically length-dependent sensory or sensorimotor polyneuropathy, accompanied with various degrees of autonomic neuropathy, (2) frequent bilateral median neuropathies at the wrist (CTS), occurring concurrently or precedingly to polyneuropathy, (3) insidious onset of sensory and/or autonomic polyneuropathy and subsequent motor polyneuropathy, and (4) progression over years to decades (faster worsening once motor nerves were affected). Additionally, symptomatic patients were not restricted to late adulthood and elderly individuals. Patients’ symptoms may start in their early 30 s. A review of the English literature in the PubMed search engine revealed 12 clinical studies with clinical data (including case reports) of ATTRA97S-PN [6–10, 17–23]. The first case was reported in 1999 [23]. Additional file 2  shows the epidemiologic data and clinical features of Thai patients in comparison to those in 12 previous reports [6–10, 17–23].

In this cohort, sensory and/or autonomic dysfunctions were reported as early symptoms and were objectively evident in the initial neurological assessments in all patients. In the report of Chao HC, autonomic symptoms were found in 68.5 % of patients during their first clinical evaluations [6]. In the study of Chao CC, most (21/28, 75 %) patients had sensory symptoms at onset [8]. In the same study, small sensory fiber loss and sudomotor nerve dysfunction were also revealed by pathological studies in 92.9 and 100 % of patients, respectively [8]. Because sensory symptoms are subjective and autonomic complaints can be nonspecific, objective evidence is inevitably important. In our study, comprehensive neurophysiological tests were used to confirm neuropathy and to grade the severity. In all four milder patients with normal NCS, the QST, which, in one test, assessed large and small sensory nerve functions, and the AFT, were abnormal, suggesting the lower sensitivity of NCS in neuropathic evaluation of ATTRv-PN, especially in the early stage [24]. Gastrointestinal dysfunctions and orthostatic intolerance were the most prevalent autonomic complaints. Gastrointestinal symptoms seemed to present in the early phase of disease, like previous studies [7, 10, 20]. Sudomotor dysfunction, though frequently evident by neurophysiological tests (the results of 7/7 QSART tested patients were abnormal.), was clinically unnoticeable in three patients. This was probably because the dysfunction was mild or mostly limited to the feet. Also, this might reflect that the sudomotor dysfunction is clinically less significant compared to gastrointestinal or cardiovascular autonomic involvement in this disease.

In contrast to other studies which recruited patients, who already had sensorimotor polyneuropathy or were the late stage [8, 9], the frequency of motor neuropathy in our study was comparably lower. This observation was because almost half of our patients were in the early stage of disease. Motor neuropathy appeared in the later stage of disease and was usually accompanied by significant weight reduction. Weight reduction has been hypothesized to be due to gastrointestinal dysautonomia, dysphagia and muscular atrophy [7]. Of interest, our study showed that when motor neuropathy was present, the overall worsening of neuropathy tended to progress faster than when motor neuropathy was not present in both clinical and neurophysiological aspects (Table 2). In the study of Yang, the time of progression from mild to significant motor dysfunctions, for which the patient required a wheelchair to ambulate, was 2.5 to 8 years [9]. In four patients with preserved motor nerves, clinical worsening was subtle, and the neurophysiological studies showed no significant change in the follow-up time up to four years. The mean annual rate of change in the NIS in our patients with motor neuropathy was also comparable to that found in a meta-analysis of ATTRv-PN (11.7 points/year) [25]. Long-term follow-up is necessary to clearly show the natural history of ATTRA97S-PN. Diflunisal is the only disease-modifying drug available in Thailand. Three patients had long-term diflunisal treatment with good tolerability. The remaining patients did not receive diflunisal. This was due to patients’ decisions (Two developed gastritis-related symptoms after treatment and decided to stop.).

In ATTRA97S-PN, concurrent cardiopathy was not unusual [16]. It was also the leading cause of death in this cohort. Cardiac manifestations included progressive heart failure, conduction defects, arrhythmias, and sudden cardiac arrest. In ATTRA97S, cardiac involvement may be asymptomatic or pauci-symptomatic, at the time neuropathy is clearly evidenced. Cardiopathy preceding neuropathy was comparatively rarer [16]. In other mutations, such as ATTRV122I and ATTRT60A, cardiopathy may precede neuropathy or be a core clinical feature [12]. Systemic involvement, such as central nervous system, eye, and kidney involvement, was also uncommon in ATTRA97S. Concurrent monoclonal gammopathy could lead to misdiagnosis of paraproteinemic neuropathies or light-chain amyloidosis, potentially delaying the diagnosis [5]. In this study, serum/urine immunofixations were also done in all patients. Only one patient had monoclonal gammopathy. This patient was initially misdiagnosed with paraproteinemic neuropathy. In this case, bone marrow biopsy was performed to exclude plasma cell dyscrasia and light-chain amyloidosis. Because all patients had a positive family history, most bypassed tissue biopsy and underwent genetic tests.

Of interest, the age of onset appeared lower in this cohort and neuropathy can be evident in middle adulthood. ATTRA97S-PN has usually been found to manifest with late-onset (> 50 years) polyneuropathy [4, 6, 7]. However, two reported patients from mainland China had early-onset (38 and 23 years) disease [18, 19]. Both carried a heterozygous variant. One patient had slowly progressive autonomic neuropathy for two decades before developing muscular weakness at 58 years [19]. Another patient had had autonomic neuropathy at 23 and developed sensory and motor polyneuropathy two years later [18]. In our study, 3/9 patients had neuropathy starting in their early 30 s. The neuropathy of three patients manifested in the form of sensory and/or autonomic neuropathy. The course of disease during their 30 s (and until the 40 s in patient D1) seemed to be protracted without motor involvement. In two patients, whose parents were also examined, the age of onset was at least 20 years lower in the younger generation. In ATTRV30M, diversity in the age of onset among different populations is a recognized phenomenon [5, 26]. Anticipation (younger onset in the offspring, usually with increased severity) has also been repeatedly reported in ATTRV30M but is not common in ATTRA97S [18, 27–31]. Similar to patients from Malaysia [21], the paternal or maternal ancestors of our patients migrated from southern China in the 19th century. It is possible that ATTRA97S-PN patients residing in Southeast Asian countries share a similar ancestor with Han-Taiwanese/Han-Chinese patients. Hence, these may reflect the common founder among those patients, rather than “hot-spot” mutation. Another supportive reason was that this mutation has not been reported in non-Han Chinese-related populations (excluding some cases with undocumented ethnicity). Further studies should be carried out to confirm this hypothesis. Population and genetic admixtures as well as environmental changes may contribute to modifying some phenotypes in Thai patients. However, whether anticipation is a biological phenomenon in ATTRA97S-PN still needs more evidence.

The treatment of ATTRv has expanded significantly during recent years, from symptomatic treatments and liver transplantation to increasing numbers of approved pharmacological options and their increasing availability. For current medications, research has emphasized the benefits of early treatment, prior to amyloid accumulation in tissues. Because the clinical manifestations of ATTRv-PN vary, diagnosis may not always be straightforward. In early onset ATTRV30M-PN, autonomic symptoms are prominent, resulting in easier recognition [5]. In ATTRA97S-PN, patients can present in their 30 s with relatively milder autonomic or sensory symptoms. As a result, physicians may overlook those apparently nonspecific autonomic complaints, particularly when some investigations were also negative. In such cases, we stress the importance of careful history taking, including a family history and detailed physical examination. In clinically suspicious cases, noninvasive neurophysiological tests (QST/AFT) objectively yield valuable neuropathic assessments.

This study had some limitations. First, due to the retrospective nature, some clinical data were lacking. Some data prior to clinical assessment were based merely on patient reports. However, in the authors’ opinion, the COMPASS-31 questionnaire had helped to systematize the collected autonomic symptoms. Second, to demonstrate the natural history of disease, a longer follow-up time is better. Diflunisal, a TTR tetramer stabilizer, was also given to three patients with motor nerve involvement, which may have modified the natural history of disease. Third, this study mainly focused on neuropathy, and less data on cardiopathy were discussed. Additionally, myocardial scintigraphy with the bone avid tracer ^99m^technetium pyrophosphate, which has high sensitivity and specificity in the diagnosis of cardiac amyloidosis, was performed in only one case [12]. Finally, this study did not evaluate the quality of life, which could reflect the impact of disease.

## Conclusions

This study is the first to show clinical and serial comprehensive neurophysiological profiles of ATTRA97S-PN in a Thai cohort. Most patients had mixed polyneuropathy and cardiopathy. Autonomic and/or sensory polyneuropathy was a more frequent presentation than motor polyneuropathy, which would appear in the late stage of disease. The age of onset was comparatively lower in Thai patients. Some developed early-onset (< 40 years) and progressive disease, starting in the form of sensory and autonomic polyneuropathy. ATTRA97S-PN should also be included in the differential diagnoses of sensory and autonomic polyneuropathy in middle-aged patients. This mutation has not yet been found in populations other than Han Chinese-related populations, probably suggesting a founder effect. Further studies are warranted.

## Supplementary information

**Additional file 1**

**Additional file 2**

## Data Availability

The datasets analyzed in this study are available on reasonable request from the corresponding author. They are not openly available as they are personal data.

## Abbreviations

ATTRv: Hereditary transthyretin amyloidosis
ATTRv-PN: ATTRv-associated polyneuropathy
TTRV30M: *p. Val50Met* in *TTR* gene
TTRA97S: *p. Ala117Ser* in *TTR* gene
ATTRA97S-PN: Ala97Ser transthyretin amyloidosis-associated polyneuropathy
NCS: Nerve conduction study
EMG: Electromyography
QST: Quantitative sensory test
AFT: Autonomic function test
COMPASS-31: Composite autonomic symptom score-31
VDT: Vibratory detection threshold
CDT: Cold detection threshold
HPS: Heat pain sensation
QSART: Quantitative sudomotor axonal reflex test
CASS: Composite autonomic severity score
CTS: Carpal tunnel syndrome
MGUS: Monoclonal gammopathy of undetermined significance
FAP: Familial amyloid polyneuropathy
ATTRV30M: Val30Met hereditary transthyretin amyloidosis
ATTRA97S: Ala97Ser hereditary transthyretin amyloidosis
ATTRV112I: Val112Ile hereditary transthyretin amyloidosis
ATTRT60A: Thr60Ala hereditary transthyretin amyloidosis
