# Supplementary material for: Ala97Ser transthyretin amyloidosis-associated polyneuropathy, clinical and neurophysiological profiles in a Thai cohort
Source: BMC Neurol. 2021 May 22;21:206. doi: 10.1186/s12883-021-02243-3 (PMC8140461; doi:10.1186/s12883-021-02243-3)
Supplement: Supplementary file 1 — Additional file 1 [file 12883_2021_2243_MOESM1_ESM.docx]

**Title:**

**Ala97Ser transthyretin amyloidosis-associated polyneuropathy, clinical and neurophysiological profiles in a Thai cohort.**

**Authors:**

Nath Pasutharnchat, MD 1,2            Email: [nathpasu@hotmail.com](mailto:nathpasu@hotmail.com), nath.p@chula.ac.th

Chamaiporn Taychargumpoo, MD 3        Email: cooksycooksy@hotmail.com

Yongkasem Vorasettakarnkij, MD  4       Email: [yongkasem@yahoo.com](mailto:yongkasem@yahoo.com)

Jakkrit Amornvit, MD 1,2                Email: jakkrit.a@chula.ac.th

Authors’ institutional affiliations:

1. Division of Neurology, Department of Medicine, Faculty of Medicine, Chulalongkorn University, Bangkok, Thailand

2. King Chulalongkorn Memorial Hospital, Thai Red Cross Society, Bangkok, Thailand

3. Hospital for Tropical diseases, Faculty of Tropical Medicine, Mahidol University, Bangkok, Thailand

4. Department of Medicine, Faculty of Medicine, Chulalongkorn University, Bangkok, Thailand

Address: Division of Neurology, King Chulalongkorn Memorial Hospital, Bangkok, Thailand 10330.

Phone: (+66)2256400 Ext 80723

Fax: (+66) 2256400Ext 80722

Corresponding author:

Nath Pasutharnchat, MD            Email: nathpasu@hotmail.com

Address: Division of Neurology, Department of Medicine, Faculty of Medicine, Chulalongkorn University, and King Chulalongkorn Memorial Hospital, Thai Red Cross Society, Bangkok, Thailand 10330.

Phone: (+66)2256-4000 Ext 80723

Fax: (+66) 2256-4000 Ext 80722

Additional file 1

Protocols of NCS included 1, Sensory nerve conduction study: bilateral median (wrist-digit II), bilateral ulnar (wrist-digit V), bilateral superficial radial (forearm-wrist) and bilateral sural (calf-ankle) and 2, Motor nerve conduction study: bilateral median- abductor pollicis brevis (APB), bilateral ulnar- abductor digiti minimi (ADM), bilateral common fibular-extensor digitorum brevis (EDB) and bilateral tibial-AH (abductor hallucis) nerve-muscle pairs. Median and ulnar motor nerve conduction studies recording over the second lumbrical and first palmar interosseous may also be applied to evaluate median neuropathy at the wrist. EMG was done in one distal and one intermediate or proximal muscles of upper and lower limbs in the non-dominant side, and in the APB if carpal tunnel syndrome was suspected by NCS.
