# Supplementary material for: Ala97Ser transthyretin amyloidosis-associated polyneuropathy, clinical and neurophysiological profiles in a Thai cohort
Source: BMC Neurol. 2021 May 22;21:206. doi: 10.1186/s12883-021-02243-3 (PMC8140461; doi:10.1186/s12883-021-02243-3)
Supplement: Supplementary file 2 — Additional file 2 [file 12883_2021_2243_MOESM2_ESM.pptx]

## Slide 1
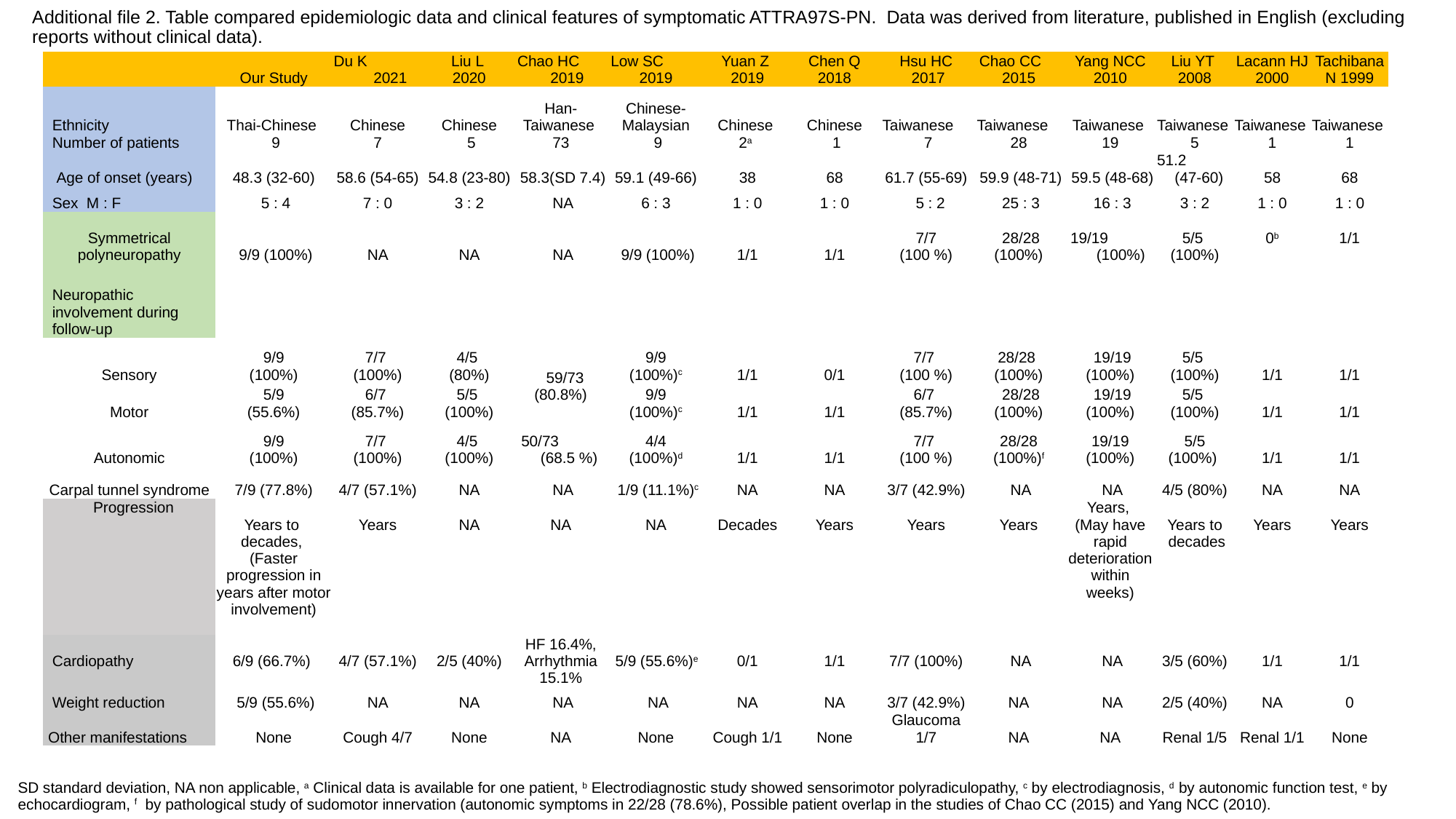

Additional file 2. Table compared epidemiologic data and clinical features of symptomatic ATTRA97S-PN. Data was derived from literature, published in English (excluding reports without clinical data).
| | Our Study | Du K 2021 | Liu L 2020 | Chao HC 2019 | Low SC 2019 | Yuan Z 2019 | Chen Q 2018 | Hsu HC 2017 | Chao CC 2015 | Yang NCC 2010 | Liu YT 2008 | Lacann HJ 2000 | Tachibana N 1999 |
| --- | --- | --- | --- | --- | --- | --- | --- | --- | --- | --- | --- | --- | --- |
| Ethnicity Number of patients | Thai-Chinese 9 | Chinese 7 | Chinese 5 | Han-Taiwanese 73 | Chinese- Malaysian 9 | Chinese 2a | Chinese 1 | Taiwanese 7 | Taiwanese 28 | Taiwanese 19 | Taiwanese 5 | Taiwanese 1 | Taiwanese 1 |
| Age of onset (years) | 48.3 (32-60) | 58.6 (54-65) | 54.8 (23-80) | 58.3(SD 7.4) | 59.1 (49-66) | 38 | 68 | 61.7 (55-69) | 59.9 (48-71) | 59.5 (48-68) | 51.2 (47-60) | 58 | 68 |
| Sex M : F | 5 : 4 | 7 : 0 | 3 : 2 | NA | 6 : 3 | 1 : 0 | 1 : 0 | 5 : 2 | 25 : 3 | 16 : 3 | 3 : 2 | 1 : 0 | 1 : 0 |
| Symmetrical polyneuropathy | 9/9 (100%) | NA | NA | NA | 9/9 (100%) | 1/1 | 1/1 | 7/7 (100 %) | 28/28 (100%) | 19/19 (100%) | 5/5 (100%) | 0b | 1/1 |
| Neuropathic involvement during follow-up | | | | | | | | | | | | | |
| Sensory | 9/9 (100%) | 7/7 (100%) | 4/5 (80%) | 59/73 (80.8%) | 9/9 (100%)c | 1/1 | 0/1 | 7/7 (100 %) | 28/28 (100%) | 19/19 (100%) | 5/5 (100%) | 1/1 | 1/1 |
| Motor | 5/9 (55.6%) | 6/7 (85.7%) | 5/5 (100%) | | 9/9 (100%)c | 1/1 | 1/1 | 6/7 (85.7%) | 28/28 (100%) | 19/19 (100%) | 5/5 (100%) | 1/1 | 1/1 |
| Autonomic | 9/9 (100%) | 7/7 (100%) | 4/5 (100%) | 50/73 (68.5 %) | 4/4 (100%)d | 1/1 | 1/1 | 7/7 (100 %) | 28/28 (100%)f | 19/19 (100%) | 5/5 (100%) | 1/1 | 1/1 |
| Carpal tunnel syndrome | 7/9 (77.8%) | 4/7 (57.1%) | NA | NA | 1/9 (11.1%)c | NA | NA | 3/7 (42.9%) | NA | NA | 4/5 (80%) | NA | NA |
| Progression | Years to decades, (Faster progression in years after motor involvement) | Years | NA | NA | NA | Decades | Years | Years | Years | Years, (May have rapid deterioration within weeks) | Years to decades | Years | Years |
| Cardiopathy | 6/9 (66.7%) | 4/7 (57.1%) | 2/5 (40%) | HF 16.4%, Arrhythmia 15.1% | 5/9 (55.6%)e | 0/1 | 1/1 | 7/7 (100%) | NA | NA | 3/5 (60%) | 1/1 | 1/1 |
| Weight reduction | 5/9 (55.6%) | NA | NA | NA | NA | NA | NA | 3/7 (42.9%) | NA | NA | 2/5 (40%) | NA | 0 |
| Other manifestations | None | Cough 4/7 | None | NA | None | Cough 1/1 | None | Glaucoma 1/7 | NA | NA | Renal 1/5 | Renal 1/1 | None |
SD standard deviation, NA non applicable, a Clinical data is available for one patient, b Electrodiagnostic study showed sensorimotor polyradiculopathy, c by electrodiagnosis, d by autonomic function test, e by echocardiogram, f by pathological study of sudomotor innervation (autonomic symptoms in 22/28 (78.6%), Possible patient overlap in the studies of Chao CC (2015) and Yang NCC (2010).
